# Awareness of Stroke Risk after TIA in Swiss General Practitioners and Hospital Physicians

**DOI:** 10.1371/journal.pone.0135885

**Published:** 2015-08-18

**Authors:** Sven Streit, Philippe Baumann, Jürgen Barth, Heinrich P. Mattle, Marcel Arnold, Claudio L. Bassetti, Damian N. Meli, Urs Fischer

**Affiliations:** 1 Institute of Primary Health Care BIHAM, University of Bern, Bern, Switzerland; 2 Institute of Complementary and Integrative Medicine, University Hospital Zurich, Zurich, Switzerland; 3 Department of Neurology, University Hospital Bern, University of Bern, Bern, Switzerland; University of Münster, GERMANY

## Abstract

**Background:**

Transient ischemic attacks (TIA) are stroke warning signs and emergency situations, and, if immediately investigated, doctors can intervene to prevent strokes. Nevertheless, many patients delay going to the doctor, and doctors might delay urgently needed investigations and preventative treatments. We set out to determine how much general practitioners (GPs) and hospital physicians (HPs) knew about stroke risk after TIA, and to measure their referral rates.

**Methods:**

We used a structured questionnaire to ask GPs and HPs in the catchment area of the University Hospital of Bern to estimate a patient’s risk of stroke after TIA. We also assessed their referral behavior. We then statistically analysed their reasons for deciding not to immediately refer patients.

**Results:**

Of the 1545 physicians, 40% (614) returned the survey. Of these, 75% (457) overestimated stroke risk within 24 hours, and 40% (245) overestimated risk within 3 months after TIA. Only 9% (53) underestimated stroke risk within 24 hours and 26% (158) underestimated risk within 3 months; 78% (473) of physicians overestimated the amount that carotid endarterectomy reduces stroke risk; 93% (543) would rigorously investigate the cause of a TIA, but only 38% (229) would refer TIA patients for urgent investigations “very often”. Physicians most commonly gave these reasons for not making emergency referrals: patient’s advanced age; patient’s preference; patient was multimorbid; and, patient needed long-term care.

**Conclusions:**

Although physicians overestimate stroke risk after TIA, their rate of emergency referral is modest, mainly because they tend not to refer multimorbid and elderly patients at the appropriate rate. Since old and frail patients benefit from urgent investigations and treatment after TIA as much as younger patients, future educational campaigns should focus on the importance of emergency evaluations for all TIA patients.

## Introduction

Transient ischemic attacks (TIA) are warning signs of stroke and require emergency treatment [1], which can prevent subsequent strokes in patients of all ages, even if they are comorbid [2]. European and American guidelines (ESO, ASA/AHA) both recommend TIA be immediately investigated, but many patients delay going to the doctor or ER, and physicians may not realize patients must be immediately referred, tested, or treated [3, 4]. Those with TIA often make first contact with family members and general practitioners (GPs), rather than with stroke specialists in emergency rooms (ER) or TIA clinics [5].

Earlier surveys showed that primary care physicians found it hard to diagnose and manage TIAs, and that they treated TIA patients less urgently than stroke victims [6–10]; almost all of these studies were based on small samples. Recently, GPs and neurologists were the target of several articles in Swiss medical journals that described how to manage patients with TIAs [11][12][13] and the problem of estimating risk after TIA was commonly discussed at local, national and international conferences. However, these measures may not have effectively alerted GPs to stroke risk after TIA, and their willingness to refer TIA patients for emergency evaluation has not yet been assessed.

We hypothesized that GPs would underestimate stroke risk after TIA, and that underestimates would largely account for failure to schedule tests or refer patients for emergency evaluation. Our goal was to determine how much Swiss GPs and hospital physicians (HPs) knew about stroke risk after TIA. We used a structured questionnaire to find out if GPs refer patients with suspected TIA for emergency evaluation, and statistically analysed physicians’ reasons for not immediately referring patients.

## Methods

### Study area and population

We invited the participation of all GPs and HPs (specialists in general internal medicine) in the populations of Bern, Lucerne, Solothurn, Obwalden and Nidwalden cantons, and the German-speaking areas of Fribourg and Wallis. In 2012, there were about 1.8 million people in our catchment area. Since there is no national registry of GPs, to identify GPs, we searched the registries of the national association of GPs (Schweizerische Gesellschaft für Allgemeinmedizin, SGAM) and the occupational union (Hausärzte Schweiz, MFE). SGAM and MFE gave us access to their database so we could contact GPs. We searched the institutional websites of all hospitals within the catchment area to identify HPs. Physicians provided their consent to participant implicit by replying the survey. The study was performed according to the ethical guidelines of the canton of Bern. An approval by an ethic committee was not required since data were non-medical and collected anonymously. The local ethic committee of Bern issued a waiver.

### Processes and Outcomes

In 2013, we sent an email invitation to all GPs and HPs (n = 1545) in the catchment area to participate in an online survey. The survey was hosted on the SurveyMonkey website (www.surveymonkey.com, Palo Alto, CA, USA), which uses IP-addresses to prevent duplicate replies. We sent non-responders two email reminders. If they still did not answer, we sent them a printed copy of the survey by postal mail. The survey (S1 Appendix) included three clinical vignettes with typical clinical pictures of patients with TIA. Vignette 1 described a typical clinical case of TIA and asked physicians to estimate, on a Likert scale, a patient’s risk of a subsequent stroke within 24 hours, and within 3 months. To make it easier for physicians to estimate risk, we listed the 1-year stroke risk of an 85-year-old man. We also asked participants how confident they were of their risk assessment. In Vignette 2, physicians were asked to select their next step after a TIA (multiple choice question). Physicians were also asked to select the aetiology of the TIA with the highest rate of recurrence (multiple choice question). In Vignette 3, participants were asked to estimate the amount by which carotid endarterectomy would reduce the risk of stroke in patients who had symptomatic carotid artery stenosis. We again asked how confident physicians were of their risk assessment. In addition to the vignettes, we also asked physicians how often they treated patients with TIA, if they investigated TIAs rigorously, if they immediately refer patients to an emergency room, and their reasons for or against immediate referral.

### Statistical analysis

We compared baseline characteristics (age, sex) using Chi-square or Fisher's exact test for categorical data, and differences in estimation of stroke risk using t-test for continuous data. We analysed GP data separately, and stratified by confidence in risk estimates, experience with TIA, and age of the physician. We dichotomized co-variables and omitted underestimates of risk so we could understand the possible confounding mechanism in overestimated risk assessments. To compare the third risk estimate (carotid endarterectomy) with the other two, we collapsed both overestimated risk reductions (40% and 50%) into one category. We calculated p-value using Chi-square test and Spearman's rho for risk estimation as the independent variable. Each of the other co-variables (experience, age, self-confidence) was used as the dependent variable to detect correlation. Finally, we performed a logistic regression analysis to assess predictors of immediate emergency referral by GPs in cases of suspected TIA. We considered a p-value of 0.05 to be statistically significant. All analysis was done with STATA release 13.1 (Stata Corp, College Station, TX, USA).

## Results

### Baseline characteristics

**Fig 1:**
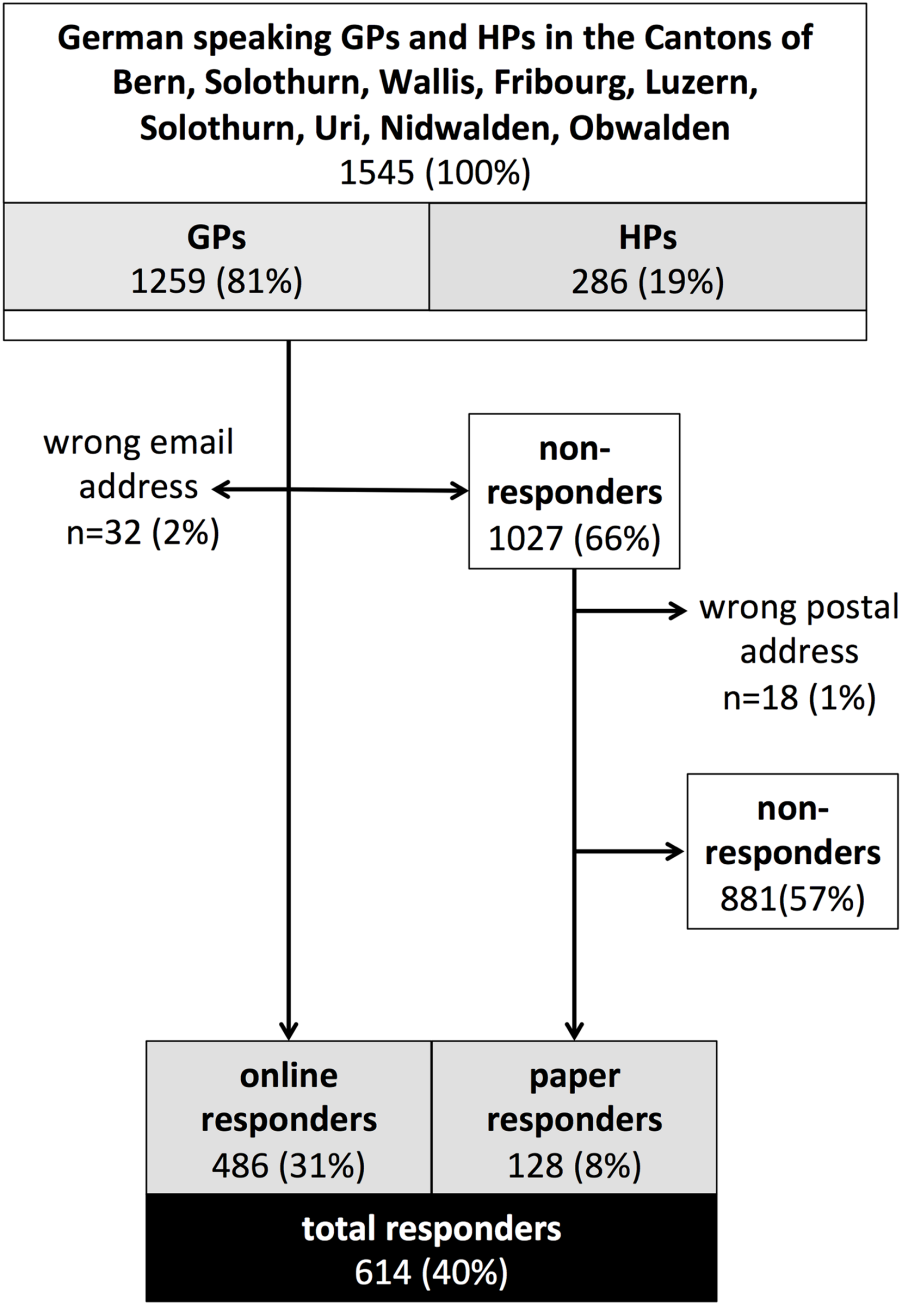
Flow Chart.

We contacted 1545 physicians (1259 GPs and 286 HPs); 40% (614) responded, 79% (486) to the online questionnaire, and 21% (129) to the postal questionnaire. Fig 1 is a flowchart of response rate. HPs were more likely to respond to the online questionnaire than GPs (87% vs. 76%). Table 1 shows baseline characteristics of GPs and HPs.

**Table 1:** Baseline characteristics of participating general practitioners (GPs) and hospital physicians (HPs).

Characteristics	Overall (n = 614)^a^	GPs (n = 470)	HPs (n = 118)	p-value
Age, mean (SD)	51.7 (9.3)	53.8 (8.4)	43.5 (8.5)	<0.001
Women, no. (%)	147 (25.1)	102 (21.8)	45 (38.1)	<0.001
Level^b^, no (%)				
Senior physician	-	-	61 (53.5)	
Attending physician	-	-	35 (30.7)	
Chief physician	-	-	18 (15.8)	
Type of office^b^, no (%)	-			
single office	-	215 (48.9)	-	
group office	-	225 (51.1)	-	
Experience with TIAs^c^, no (%)				<0.001
very rarely	67 (11.5)	56 (12)	11 (9.3)	
rarely	189 (32.3)	176 (37.8)	13 (11.0)	
sometimes	263 (45.0)	223 (47.9)	40 (33.9)	
often	54 (9.2)	10 (2.2)	43 (36.4)	
very often	12 (2.1)	1 (0.2)	11 (9.3)	

^a^26 physicians filling out the paper version of the questionnaire did not state whether they are GPs or HPs.

^b^These Characteristics where applicable to GPs only, HPs only respectively.

^c^Participants where asked: "I see patients with transient ischemic attacks (TIAs)…"

### Stroke risk estimation after TIA

Table 2 details the stroke risk estimates made by GPs and HPs. A large majority of physicians (75%, 457) overestimated risk. They were much less likely to underestimate risk of stroke in the 24 hours subsequent to TIA (9%, 53). GPs were more likely to underestimate and HPs more likely to overestimate the risk (p = 0.01). In answer to the question about stroke risk within the subsequent three months, 40% (245) overestimated, and 26% (158) underestimated; there was no difference between GPs and HPs (p = 0.6). Most physicians (78%) overestimated reduction in stroke risk in the five years after carotid endarterectomy; only 6% underestimated risk reduction; there was no difference between GPs and HPs (p = 0.14). When asked for the aetiology with the highest recurrence rate, over half of physicians (61%) incorrectly labelled cardioembolic TIAs as the most dangerous cause of stroke; 25% correctly answered a large vessel stenosis, and 11% though it was small vessel diseases (11%). GPs and HPs estimated the risk of underlying aetiology differently (p = 0.002); HPs were more likely to correctly label a large vessel stenosis to be the major cause of stroke (36% vs 22% in GPs)

**Table 2:** Stroke risk estimations after TIA and causes for recurrence by general practitioners and hospital physicians*.

Questions and Answers	Overall n = 614**	General Practitioner n = 470	Hospital Physician n = 118	p-value
Stroke risk estimation within 24h after TIA, n (%)				0.01
underestimated	53 (8.7)	49 (10.5)	3 (2.5)	
correctly estimated	103 (16.8)	74 (15.8)	24 (20.3)	
overestimated	457 (74.6)	346 (73.8)	91 (77.1)	
Stroke risk estimation within 3 months after TIA, n (%)				0.60
underestimated	158 (25.8)	121 (25.8)	33 (28.0)	
correctly estimated	210 (34.3)	162 (34.5)	35 (29.7)	
overestimated	245 (40.0)	186 (39.7)	50 (42.4)	
Estimation of risk reduction of stroke after carotid endarterectomy in 5 years, n (%)				0.14
underestimated	33 (5.5)	24 (5.1)	7 (5.9)	
correctly estimated	98 (16.2)	9 (14.7)	26 (22.0)	
overestimated	473 (78.3)	375 (80.1)	85 (72.0)	
Estimation of TIA cause with highest rate of recurrence, n (%)				0.002
Cardioembolic	370 (61.2)	298 (64.0)	58 (59.2)	
Small vessel disease	64 (10.6)	52 (11.2)	10 (8.5)	
Large vessel stenosis (correct answer)	150 (24.8)	103 (22.1)	42 (35.6)	
undetermined/unknown	21 (3.5)	13 (2.8)	8 (6.8)	

*for exact questions and answer possibilities see S1 Appendix.

**26 physicians did not state to be GP or HP.

### Stratifying risk estimates

In Table 3, we stratified risk estimates by physician experience in treating patients with TIA, how confident they were of their risk estimates, and the age of the physician (cut-off was 55 years). Those who were more confident of the accuracy of their risk estimate were more likely to overestimate stroke risk, and to overestimate the amount that endarterectomy reduced risk. Doctors who had more experience treating patients with TIA were not likely to be better at estimating risk; age also had no influence on risk estimates.

**Table 3:** Stratification of prediction of risk for experience with patients with TIA, self-confidence with risk estimation and physician's age.

Stratification	Stroke risk within 24 hours after TIA		Stroke risk next 3 months following TIA		Stroke risk reduction by endarterectomy	
	*p-value*	*Spearman's rho*	*p-value*	*Spearman's rho*	*p-value*	*Spearman's rho*
Experience treating patients with TIA	0.21	0.08	0.65	-0.02	0.20	-0.02
Self-confidence with stroke risk estimation	<0.001	0.21	<0.001	0.26	<0.001	0.16
Physician's age (cut off 55 years)	0.71	-0.03	0.93	0.01	0.20	0.07

### Investigational procedures

Table 4 summarizes the next diagnostic steps doctors would take if they suspected patients had TIA. Almost all physicians (93%; 543; no difference between GPs and HPs) said they would rigorously investigate the cause of a TIA. Over half (55%; 330) would immediately refer patients to the ER. GPs and HPs chose different diagnostic procedures (p = 0.017). Though 38% of physicians (229) would “very often” immediately refer patients suspected of TIA to an ER, HPs were more likely to refer than GPs (p<0.001). Physicians gave different reasons for not referring patients immediately to an ER, 144 (13%) mentioned advanced age of a patient, 138 (13%) a patient’s wish to avoid further tests, and 111 (10%) the need for long-term care, and multimorbidity (see S1 Table for all reasons against referral). Physicians would immediately refer a patient with suspected TIA to an ER if they had cardiovascular risk factors (10%) or were younger (9%). Our logistic regression analysis showed a positive association between a physician’s belief that the aetiology of a TIA should be rigorously investigated and the likelihood they would immediate referral patients with suspected TIA to ER (OR 2.0, 95% CI 1.5–2.7, p<0.001).

**Table 4:** Investigational procedures chosen by general practitioners and hospital physicians.

Questions and Answers	Overall n = 614*	General Practitioner n = 470	Hospital Physician n = 118	p-value
I investigate the causes of TIA rigorously, n (%)				0.85
very rarely	3 (0.5)	2 (0.4)	1 (0.9)	
rarely	4 (0.7)	3 (0.6)	0	
sometimes	35 (6.0)	28 (6.0)	7 (5.9)	
often	195 (33.3)	158 (33.9)	37 (31.4)	
very often	348 (59.5)	275 (59.0)	73 (61.9)	
In patients suspected for a TIA, my next step is…				0.017
Immediate admission to the ER	330 (54.6)	247 (53.1)	79 (67.0)	
MRI brain with angiography of brain supplying vessels within the next 48 hours	98 (16.2)	73 (15.7)	20 (17.0)	
MRI brain and ECG within the next 48 hours	74 (12.3)	59 (12.7)	10 (8.5)	
CT brain and 24-hours-ECG within the next 24 hours	36 (6.0)	29 (6.2)	2 (1.7)	
an other step	66 (10.9)	57 (12.3)	7 (5.9)	
In cases of suspected TIA, I would immediately refer the patient to an emergency room, n (%)				<0.001
very rarely	23 (3.8)	19 (4.1)	2 (2.6)	
rarely	39 (6.5)	37 (7.9)	0	
sometimes	98 (16.3)	89 (19.1)	5 (4.3)	
often	213 (35.4)	168 (36.0)	37 (31.6)	
very often	229 (38.0)	154 (33.0)	72 (61.5)	

*26 physicians did not state to be GP or HP.

## Discussion

We found that physicians overestimate risk of stroke within 24 hours and within three months of TIA, and they overestimate how much carotid endarterectomy lowers the risk of stroke. More than 90% of physicians say they would rigorously investigate the cause of a TIA, but in results of the vignettes and general questions indicated that only half of them would immediately refer patients to an ER for further work-up. The main reasons physicians did not immediately refer patients included a patient’s need for long-term care, multimorbidity, patient desire to avoid further tests, and that the patient was very old.

Earlier studies found that a third of patients diagnosed with TIA in primary care clinics were not hospitalized and did not receive further tests or treatment [8]. Studies from Japan, France, Poland, Australia and the United States affirmed that most physicians are undereducated about the risk of stroke after TIA; many found it difficult to manage these patients [6, 7, 9, 10, 14]. It was not only primary care physicians who lacked knowledge; neurologists had the same problem [9].

In contrast to these studies, and counter to our hypothesis, we found that Swiss physicians tended to overestimate the risk of stroke after TIA, perhaps because there has been a lot of effort, in Switzerland, to raise awareness of that risk. This may be why less than 10% of physicians underestimated stroke risk with 24 hours after TIA, and less than 30% underestimated risk within 3 months. Most physicians (78%) also overestimated the benefits of carotid endarterectomy on reducing stroke. Physicians commonly overrate the benefits of surgical procedures [15].

Contrary to European and American guidelines (ESO, ASA/AHA) that recommend investigating TIA within the first 24 hours, and despite their overestimates of stroke risk and the benefits of therapy after TIA, only 55% of physicians would immediately refer their patients to an ER. Even fewer (45%) would schedule tests within two days of the event for patients suspected of TIA. The reasons Swiss physicians usually gave for not making an immediate referral to an ER were generally inadequate. If a patient is already in palliative care, there may be a good reason not to investigate further; if they have severe dementia, the question might be debatable. But the benefit of emergency treatment for TIA is clear, even for older and multimorbid patients: over a third of the study population of two large studies (Early Use of Existing PREventive Strategies for Stroke [EXPRESS] and the population-based Oxford Vascular Study [OXVASC]) was over 80 years old and had multiple comorbidities. In the EXPRESS study, risk was reduced independent of age, so advanced age and comorbidities are not good reasons for failing to schedule TIA patients for emergency evaluation.

Our survey is limited by the relatively low response rate; only 40% (614) of 1545 physicians answered our questionnaire. We did try to increase the response rate by having medical authorities send a letter of recommendation that asked physicians to participate in the survey. We also sent reminders by email, and a final reminder by postal mail. We were also limited by our use of open-ended questions, since, for example, we could not determine why physicians considered multimorbidity a reason not to refer. On the other hand, our study was strengthened by its population-based nature, its large sample size, and its inclusion of both general and hospital physicians.

Swiss physicians overestimate stroke risk after TIA, but refer elderly and comorbid patients to the ER at a much lower rate than guidelines recommend. Educational campaigns might be more effective if they emphasize the importance of emergency management of all TIA patients, regardless of age or comorbidity.

## Supporting Information

S1 Appendix Questionnaire. (PDF)

S1 Table All reasons for physicians against immediate referral of patients suspected for TIA to an emergency ward. (DOCX)
