# Supplementary material for: Awareness of Stroke Risk after TIA in Swiss General Practitioners and Hospital Physicians
Source: PLoS One. 2015 Aug 18;10(8):e0135885. doi: 10.1371/journal.pone.0135885 (PMC4540278; doi:10.1371/journal.pone.0135885)
Supplement: S1 Appendix — (PDF) [file pone.0135885.s001.pdf]

NEUROZENTRUM
Neurocentre | Neurocentro
Inselspital Universitätsspital Bern
Universitäre Psychiatrische Dienste Bern

Neurochirurgie
Neurologie
Neuropädiatrie
Neuroradiologie
Psychiatrie

BIHAM

Stroke risk after TIA – Risk estimations from General and Hospital Practitioners

FAX BIHAM: 031 632 89 90

The aim of this questionnaire is to ask you, as a General or Hospital practitioner, about the risk for stroke after a TIA, as well as risk reduction and the referral behaviour.

Three clinical vignettes:

Vignette 1

A 67-year old, previously healthy woman consults you around 10am. She reports a weakness in her left arm and left face, which appeared suddenly around 7am persisting till 8:30am. Since then she has been completely symptom-free. Apart from a blood pressure of 130/85mmHg, the clinical examination (neurological and internist) is considered unremarkable. You suspect a TIA.

What is your estimation of the absolute risk of this patient's suffering from a stroke after the TIA?
For comparison, an 85-year-old man has a 1-year-risk of a stroke of 1%.

How high is the risk in the next 24 hours? ◻ 1% ◻ 3% ◻ 5% ◻ 10%

How confident are you with your risk assessment?
◻ very unconfident ◻ unconfident ◻ confident ◻ very confident

How high is the risk in the next 3 month? ◻ 5% ◻ 10% ◻ 15% ◻ 20%

How confident are you with your risk assessment?
◻ very unconfident ◻ unconfident ◻ confident ◻ very confident

Vignette 2

A 65-year-old, previously healthy man consults you on a Wednesday, around 10am. He tells you that he could not talk that same morning. The complaints appeared abruptly around 7am and persisted for roughly 15 minutes. Since then, he has been completely symptom-free. The vital signs at the clinical examination are unremarkable. You suspect a TIA.

Choose your next step
◻ MRI brain and ECG within the next 48 hours
◻ Immediate admission to an ER
◻ MRI brain with angiography of brain supplying vessels within the next 48 hours
◻ CT brain and 24-hours-ECG within the next 24 hours
◻ Other step(s) ____

What cause of TIA has the highest rate of recurrence?
◻ Cardioembolic
◻ Small vessel disease
◻ Large vessel stenosis
◻ undetermined/unknown

Vignette 3

A 56-year-old patient, who is diabetic but otherwise healthy, consults you around 10pm. He tells you that he felt a slight weakness the right side of his body that same morning (he could hardly hold coffee cup). The complaints appeared suddenly after he woke up at around 7am and persisted for about 45 minutes. Since then, he has been completely symptom-free. The vital sings are unremarkable apart from a blood pressure of 160/95mmHg. The postprandial blood glucose (9.4 mmol/l) and the neurological examination are normal. You suspect a TIA. The investigations reveal a stenosis of about 80% of the left internal carotid artery.

How high do you estimate the risk reduction in % by carotid endarterectomy in relation to a recurrent stroke or death in 5 years? ◻ 5% ◻ 15% ◻ 30% ◻ 40% ◻ 50%

How confident are you with your risk assessment?
◻ very unconfident ◻ unconfident ◻ confident ◻ very confident

NEUROZENTRUM
Neurocentre | Neurocentro
Inselspital Universitätsspital Bern
Universitäre Psychiatrische Dienste Bern
Neurochirurgie
Neurologie
Neuropädiatrie
Neuroradiologie
Psychiatrie

Your experience with cases of suspected TIA:

We would like to know about your experience in handling patients with suspected TIA.

In cases of suspected TIA, I would immediately refer the patient to an emergency room
□ very rarely □ rarely □ sometimes □ often □ very often

Please write down circumstances / facts / reasons for or against a transferral (to an emergency room with 24-hour neuroimaging)

The following facts would motivate myself to refer urgently

1.
2.
3.
4.
5.

The following facts would motivate myself against referring urgently

1.
2.
3.
4.
5.

Personal questions:

Age: ___________ Gender: □ female □ male □ other

Role: □General Practitioner
□ single office □ group office
□ Hospital Practitioner
□ senior physician □ attending physician □ chief of medicine

I see patients with a TIA
□ very rarely □ rarely □ sometimes □ often □ very often

I investigate the causes of TIA rigorously
□ very rarely □ rarely □ sometimes □ often □ very often

Thank you you for your participation.

Please fax the questionnaire to:

FAX BIHAM: 031 632 89 90

Would you like more information?
Please let us know your e-mail adress to participate in the lucky draw for an iPad mini® and/or to get the results of the study or the answers of the questions above.

Your e-mail adress: ____ for □ answers □ lucky draw □ results
