# Supplementary material for: Awareness of Stroke Risk after TIA in Swiss General Practitioners and Hospital Physicians
Source: PLoS One. 2015 Aug 18;10(8):e0135885. doi: 10.1371/journal.pone.0135885 (PMC4540278; doi:10.1371/journal.pone.0135885)
Supplement: S1 Table — (DOCX) [file pone.0135885.s002.docx]

**S1 Supplemental Table**

Reasons for physicians not to admit a patient suspected for a TIA to an emergency ward.

| **Reasons not to admit** | **all answers (n=1098)** |
| --- | --- |
|  |  |
| **Age, general conditions** |  |
| Age >60 | 144 (13.1) |
| Patients’ wish | 138 (12.6) |
| Depending on constant care, nursing home and multimorbidity | 111 (10.1) |
| Palliative care | 67 (6.1) |
| Comorbidities | 32 (2.9) |
| Possibility to perform investigations in the outpatient clinic | 31 (2.8) |
| No therapeutic consequences estimated | 29 (2.6) |
| Unspecific symptoms | 27 (2.5) |
| Dementia | 26 (2.4) |
| No comorbidity | 18 (1.6) |
| Age <60 | 16 (1.5) |
|  |  |
| **Neurological** |  |
| No neurological symptoms during consultation | 41 (3.7) |
| Recurrent TIA with previous investigations | 40 (3.6) |
| Short duration (<15 minutes) | 21 (1.9) |
| ABCD2 score < 2 | 9 (0.8) |
| Known etiology | 8 (0.8) |
| First TIA | 8 (0.8) |
|  |  |
| **Cardiovascular** |  |
| Low cardiovascular risk profile | 50 (4.6) |
| Already on antiplatelet drugs | 15 (1.4) |
| Oral anticoagulation | 9 (0.1) |
| Not new arrhythmia | 4 (0.4) |
| Known atrial fibrillation | 1 (0.1) |
|  |  |
| **Systemic** |  |
| Hypoglycaemia | 2 (0.2) |
|  |  |
| **Other reasons** | 249 (22.7) |
|  |  |
